# Techno-economic assessment at full scale of a biogas refinery plant receiving nitrogen rich feedstock and producing renewable energy and biobased fertilisers

**DOI:** 10.1016/j.jclepro.2021.127408

**Published:** 2021-07-25

**Authors:** C. Brienza, I. Sigurnjak, T. Meier, E. Michels, F. Adani, O. Schoumans, C. Vaneeckhaute, E. Meers

**Affiliations:** a Green Chemistry and Technology Department, Faculty of Bioscience Engineering, Ghent University, Coupure Links 653, B-9000, Ghent, Belgium; b GNS - Gesellschaft für Nachhaltige Stoffnutzung mbH, Weinbergweg 23, 06120, Halle, Germany; c Gruppo Ricicla, Dipartimento di Science Agrarie e Ambientali: Produzione, Territorio, Agroenergia, Università degli Studi di Milano, Via Celoria 2, Milano, 20133, Italy; d Wageningen Environmental Research, PO Box 47, 6700, AA, Wageningen, the Netherlands; e BioEngine – Research Team on Green Process Engineering and Biorefineries, Chemical Engineering Department, Université Laval, 1065 Ave. de la Médecine, Québec, QC, G1V 0A6, Canada

**Keywords:** Circular biobased economy, Anaerobic digestion, Nutrient recovery, Ammonia stripping, Ammonium sulphate

## Abstract

Anaerobic digestion of nitrogen (N) rich substrates might be hindered when ammonia (NH_3_) formation reaches toxic levels for methanogenic microorganisms. One possible strategy to avoid inhibiting conditions is the removal of NH_3_ from digestate by stripping and scrubbing technology and by recirculating N depleted digestate back to the digester. This study aimed to i) monitor the performance (mass and energy balances) of a full scale digestate processing cascade that includes an innovative vacuum side stream NH_3_stripping and scrubbing system, ii) assess the production cost of ammonium sulphate (AS) solution and iii) evaluate its fertiliser quality. The use of gypsum to recover NH_3_ in the scrubbing unit, instead of the more common sulphuric acid, results in the generation of AS and a fertilising liming substrate. Mass and nutrient balances indicated that 57% and 7.5% of ammonium N contained in digestate was recovered in the form of a 22% AS and liming substrate, respectively. The energy balance showed that about 3.8 kWh_el_ and 59 kWh_th_ were necessary to recover 1 kg of N in the form of AS. Furthermore, the production cost of AS, including both capital and operational costs, resulted to be 5.8 € t^−1^ of digestate processed. According to the fertiliser quality assessment, this technology allows for the recovery of NH_3_in the form of salt solutions that can be utilised as a substitute for synthetic mineral nitrogen fertilisers.

## Highlights

• A novel ammonia stripping process was monitored at a fullscale digestate processing facility.
• 57% of ammonium nitrogen contained in digestate was recovered as ammonium sulphate (22%).
• Nitrogen in recovered ammonium sulphate is present entirely in mineral form.
• The cost for the nitrogen recovery system amounts to 5.8 € per tonne of digestate processed.
• Biobased ammonium sulphate represents an interesting alternative to synthetic nitrogen fertilisers.

## 1 Introduction

Interest in renewable energy is growing in the European Union (EU), resulting in the Renewable Energy Directive EU 2001/2018 (REDII), 2018. Anaerobic digestion (AD) is a promising technology as it combines the production of biogas and the recycling of nutrients in the form of an organic fertiliser known as digestate. In some European countries, the biogas production has remarkably increased in the last decade, due to the introduction of conspicuous incentives for the production of biogas from renewable biomass. In Germany, for example, the Renewable Energy Sources Act (EEG, 2000) has boosted the expansion of biogas plants and since 2010, Germany covers 50% of the European biogas production, mainly from silage maize (Meyer et al., 2018). Following the amendments of the Renewable Energy Sources Act, a dedicated bonus for biogas plants using at least 30% of manure as feedstock was introduced in 2009. As such, anaerobic co-digestion (co-AD) of silage maize and manure is an interesting option for biogas plants.

Anaerobic fermentation of nitrogen (N) rich feedstock (such as chicken manure) leads to the formation of high concentrations of ammonia (NH_3_) in the digester which can have an inhibiting effect on methanogenic microorganisms when reaching toxic levels, causing in turn failure of the AD process (Yenigün and Demirel, 2013). Many strategies have been reported in the literature to circumvent NH_3_ inhibition (Rajagopal et al., 2013; Jiang et al., 2019). These includes i) acclimatisation of digester microflora by gradual increment of NH_3_ concentrations, ii) pH and temperature control, iii) dilution of N rich feedstock with water or a co-substrate to adjust the C:N ratio, iv) immobilisation of microorganisms on inert packing material, v) supplement of trace elements to ensure methanogens biological activity vi) physico-chemical and chemical NH_3_ removal (e.g. NH_3_ stripping and scrubbing). Differently from other strategies, stripping and scrubbing and recirculation of stripped digestate allows to simultaneously remove NH_3_ from digester, thus bypassing microflora inhibition, and generate ammonium (NH_4_) salts. These products are suitable building blocks for the production of N mineral fertilisers or other chemicals (Brienza et al., 2020).

NH_3_ stripping is a gas-liquid mass transfer process where NH_3_ is stripped from the treated substrate to a gas phase, usually by air, steam, biogas or combined heat and power (CHP) flue gas (Bousek et al., 2016). The removal efficiency of NH_3_ is mainly influenced by pH, alkalinity, temperature, air to liquid ratio, air supply rate and hydraulic retention time (Gustin and Marinsek-Logar, 2011; Campos et al., 2013). Recirculation of N stripped digestate (lowered in NH_3_ content) to the AD reactor can lower the reactor concentration of NH_3_ despite high N concentration in the feedstock (Zhang et al., 2017). Next, in the scrubbing column, NH_3_ contained in the gas phase is stabilised by contact with a solution that results in the formation of so-called NH_4_ salts or scrubbing salts. At pilot and full scale installations, three different scrubbing media have proven to be effective for NH_3_ recovery: sulphuric acid solution (H_2_SO_4_), nitric acid solution (HNO_3_) and solid dihydrate calcium sulphate (gypsum, CaSO_4_.2H_2_O) (Ledda et al.; 2013, Bolzonella et al.; 2018, Sigurnjak et al., 2019). If H_2_SO_4_ or HNO_3_ are used, ammonium sulphate ((NH_4_)_2_SO_4_) or ammonium nitrate (NH_4_NO_3_) salts will be respectively formed; whereas the use of CaSO_4_.2H_2_O would result in a mixture of ammonium sulphate and liming fertiliser substrate (a mixture of calcium carbonate and gypsum) (Brienza et al., 2020).

NH_3_ stripping and subsequent scrubbing is one of the most commonly commercially available technologies on the market to remove N contained in effluents of different origins. Nevertheless, less is known about the fertiliser potential of the generated biobased ammonium sulphate from stripping and scrubbing and its impact on soil and crop production. Up to now, performance of ammonium sulphate from air scrubbers (installed on animal stables) has been assessed on lettuce (pot and greenhouse experiments) and maize (field trials). Results from different studies indicated similar dry weight (DW) and fresh weight (FW) crop yields in comparison with a conventional fertilisation regime (Sigurnjak et al., 2016). In a 2-year field trial, Vaneeckhaute et al. (2014) recorded a higher N uptake in treatments with recovered ammonium sulphate as compared to conventional mineral N fertiliser. In regards with the environmental aspects of the ammonium sulphate use, postharvest nitrate (NO_3_–N) residue analyses in maize field trials indicated similar results for both ammonium sulphate application and mineral N fertilisation. In all cases, NO_3_–N residues after fertilisation with ammonium sulphate were below the maximum allowable limits that are implemented in Flemish legislation (Vaneeckhaute et al., 2014; Sigurnjak et al., 2019).

To promote the development of a circular biobased economy and stimulate the recycling of manure-derived N, a deep understanding of digestate processing installations and a science based characterisation of recovered scrubbing salts is required. In this perspective, the implementation of mathematical models represents an added value to predict the performances of nutrient recovery technologies and quality of recycled fertilisers (Vaneeckhaute et al., 2018).

Currently, the market implementation of manure-derived scrubbing salts is hampered by the Nitrates Directive (EU) 676/1991, which identifies all manure derivatives as animal manure and limits their application on agricultural land in nitrate vulnerable zones (NVZs) to 170 kg N ha^−1^ y^−1^. In this blurred legislative background, the SAFEMANURE project (Huygens et al., 2020), led by the European Commission's Joint Research Centre (JRC), has the objective to define harmonised criteria that could allow manure-derived N fertilisers (RENURE products) to be applied in NVZs, following the same specifications of synthetic (fossil resource based) N fertilisers. More specifically, the report compiled by Huygens et al. (2020) indicates that RENURE (REcovered Nitrogen form manURE) products must have a total organic carbon:total N (TOC:TN) ratio ≤ 3 or a mineral N:TN ratio ≥ 90%. Moreover, the content of copper (Cu) and zinc (Zn) should not exceed respectively 300 mg kg^−1^ DW and 800 mg kg^−1^ DW. Despite being considered essential plant micronutrients, high application rates of Cu and Zn may have detrimental effects on soil microorganisms and crops. Often, Cu and Zn are included as additives to livestock diets as growth promoters and antibiotics alternatives (Yazdankhah et al., 2014) and up to 90% of Cu and Zn fed to animals is excreted in faeces, leading to accumulation when manure is applied in agricultural lands as fertiliser (Berenguer et al., 2008). Thus, RENURE products will be regulated for their Cu and Zn content to prevent the build-up in soil and consequent risks for the food chain. Finally, the recently proposed Fertilising Product Regulation (EU) 1009/2019 will include products partially or entirely derived from manure origin. Albeit scrubbing salts recycled from manure digestate would meet all criteria to be classified as a liquid organomineral fertiliser they may meet restrictions on land applicability due to their animal manure status in accordance with the Regulation (EU) 142/2011.

**Fig. 1:**
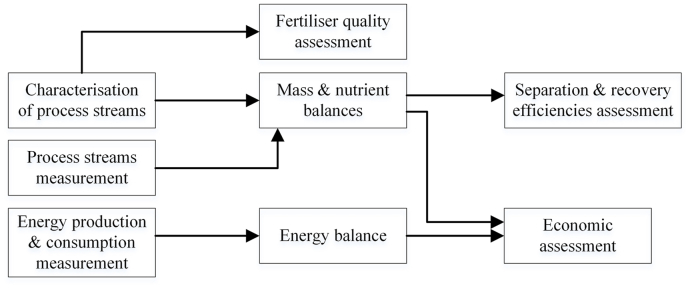
Schematic flow chart of the proposed study.

The objective of this study was to verify the performance of a full scale digestate processing installation where NH_3_ stripping and subsequent recovery with gypsum, generated a biobased ammonium sulphate solution (22%). The technological assessment was performed in terms of mass, nutrient and energy balances to evaluate the separation and recovery efficiencies and the energy requirements of the different process units. The study also includes an economic analysis of the generated ammonium sulphate solution, and an assessment of its fertilising quality with reference to the Product Function Categories (PFC) criteria of the Fertilising Product Regulation and RENURE products (Fig. 1).

## 2 Materials & methods

### 2.1 Benas site description

**Fig. 2:**
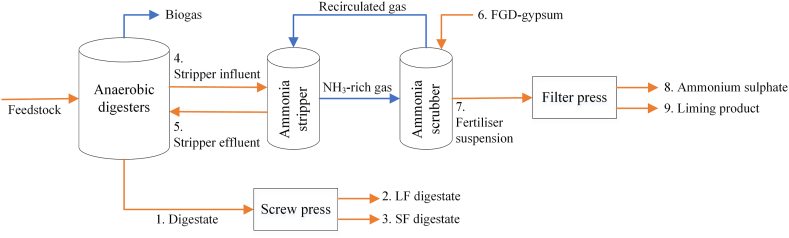
Simplified scheme of Benas digestate processing cascade. Sampling points are indicated with numbers: 1. Digestate, 2. Liquid fraction (LF) digestate, 3. Solid fraction (SF) digestate, 4. Stripper influent, 5. Stripper effluent, 6. FGD-gypsum, 7. Fertiliser suspension, 8. Ammonium sulphate, 9. Liming substrate. Gas flows are indicated in blue; solid and liquid flows are indicated in orange.

The Benas farm (Ottersberg, Germany) consists of 3,500 ha (ha) of arable land, of which 1,000 ha nearby the farm and 2,500 ha 200 km away in Saxony-Anhalt. The farm has its own co-AD installation (maximum capacity of 174,000 t y^−1^) that includes four digesters (and three storage tanks for post digestion) operated at thermophilic regime with a retention time of about 60 days. In 2019, about 41,000 t of corn silage, 28,700 t of rye silage and 13,300 t of chicken manure was used as feedstock. Other agricultural substrates included corn, millet and grass (8,900 t) and goose manure (249 t). Generated biogas (222 Nm^3^ t^−1^ feedstock) is fed to CHP engines for maximum electricity production of 11.3 MW or upgraded to biomethane. In the co-AD and digestate processing installation (Fig. 2), raw digestate (stripper influent) is treated in a side stream vacuum NH_3_ stripping and scrubbing unit, consisting of three stripping columns. In the stripping columns, digestate is heated up to 90 °C and the process does not require any external heat source, relying entirely on the exhaust heat generated by three CHP engines. Pressure is firstly brought to 0.1–0.3 bar and successively increased to 0.4–0.8 bar. CO_2_ and NH_3_ escape the system and the removal of CO_2_ fosters an increase in pH of the processed stream. The gas phase is cooled down and introduced into an aqueous absorption agent, dihydrate gypsum from a flue gas desulphurization plant (FGD-gypsum), to form a fertiliser suspension containing ammonium sulphate and liming product. Ammonium sulphate and the liming product are subsequently separated by means of a filter press. N depleted digestate (stripper effluent) is recirculated back to the co-AD to dilute N rich feedstock. The co-AD plant of Benas is also implemented with a screw press for digestate mechanical separation into a liquid and a solid fraction, from now on indicated as LF digestate and SF digestate.

A full scale digestate nutrient recovery and reuse processing cascade was monitored for four months, from January until April 2019, which corresponds to two hydraulic retention times. Over the monitoring period, the input feed of the digester amounted to 236 t d^−1^ and included corn silage (62%), chicken manure (28%), agricultural substrates (corn grain, millet and grass silage; 9%) and goose manure (1%).

### 2.2 Sampling and physico-chemical analysis of process streams

Digestate, FGD-gypsum, intermediate (stripper influent and effluent, fertiliser suspension) and final products (LF and SF digestate, ammonium sulphate and liming substrate) originated from the treatment cascade were sampled monthly during the four months monitoring period. Samples were stored in 2 L polyethylene bottles at 4 °C and subsequently analysed in replicates. pH and electrical conductivity (EC) were respectively measured using an Orion-520A pH meter (USA) and a WTW-LF537 (DE) conductivity electrode. pH and EC of solid samples were determined in a 1:5 (w w^−1^) suspension of wet solids to deionised water ratio (Van Ranst et al., 1999). DW content was determined as the residual weight after 24 h drying at 105 °C. Organic matter (OM) was determined as the weight loss of dry samples after incineration at 550 °C for 4 h in a muffle furnace (Nabertherm, Lilientahl, DE). TN was determined using Kjeldahl destruction. Ammonium N (NH_4_–N) was determined using a Kjeltec-1002 distilling unit (Gerhardt Vapodest, DE) after addition of MgO to the sample and subsequent titration with 0.01 mol HCl L^−1^ in the presence of methyl red bromocresol green mixed indicator (Van Ranst et al., 1999). Total phosphorus (TP), potassium (TK), sulphur (TS), calcium (TCa), magnesium (TMg) and sodium (TNa) were detected using Inductively Coupled Plasma - Optical Emission Spectrometry (ICP-OES Varian Vista MPX, USA), after wet digestion of ammonium sulphate (2 ml of 65% HNO_3_ + 1 ml of H_2_O_2_) and microwave digestion (10 ml 65% HNO_3_) of all other collected samples (Van Ranst et al., 1999). Cu and Zn were determined in the same manner, but only on ammonium sulphate (Van Ranst et al., 1999). The determination of TOC was determined by using TOC analyzer (TOC-5000, Shimadzu, JP).

Product composition of ammonium sulphate solution was assessed from April 2017 until March 2020 for a total of 10 sampling rounds.

### 2.3 Assessment of separation and recovery efficiencies

Separation efficiency of the screw press and filter press (Eq. (1)) stands for the mass of solids respectively in the solid and liquid fraction as a proportion of the total input of solids, and is presented as follows (Svarovsky, 1985):

(1) Se = (X * Cx) / (Y * Cy)

where Se is the separation efficiency; X (kg) the quantity of the outgoing fractions; Cx (g kg^−1^ FW) the concentration of the component (DW, TN, NH_4_–N, TP, TK, TS, TCa, TMg, TNa) in the outgoing fractions; Y (kg) the amount of ingoing substrate (i.e. digestate for the screw press and fertiliser suspension for the filter press) and Cy (g kg^−1^ FW) is the concentration of the considered component of the ingoing substrate.

Recovery efficiency of the NH_3_ stripping and scrubbing system (Eq. (2)) stands for mass recovery of TN and NH_4_–N in fertiliser suspension, ammonium sulphate and liming substrate as a proportion of the total input of solids:

(2) Re = (X * Cx) / (Y * Cy)

where Re is the recovery efficiency; X (kg) the quantity of fertiliser suspension, ammonium sulphate or liming substrate; Cx (g kg^−1^ FW) the concentration of the component (TN or NH_4_–N) in the fertiliser suspension, ammonium sulphate or liming substrate; Y (kg) the amount of ingoing substrate (stripper influent) and Cy (g kg^−1^ FW) is the concentration of the considered component (TN or NH_4_–N) in the stripper influent. Addition of TN or NH_4_–N via FGD-gypsum is negligible and therefore not included in the calculation.

Mass of water was calculated as the difference between mass flow and mass of DW. Mass of organic N (Org-N) was calculated as the difference between mass of TN and mass of NH_4_–N.

### 2.4 Assessment of energy balance

Biogas production was recorded on an industrial gas flow meter and electricity produced by the CHP was metered. The generated electrical and thermal energy were computed on a monthly basis during the entire year of 2019. The digestate processing facility was equipped to measure energy consumption. The amount of electrical and thermal energy required for the production of ammonium sulphate were computed based on the average annual measured consumptions.

### 2.5 Economic assessment

The Cost-Benefit Analyses (CBA) was determined based on the total volume of digestate and ammonium sulphate produced in 2019. The capital cost of the vacuum NH_3_ stripping and scrubbing unit and the filter press was amortised (Eq. (3)). Operational costs included electricity use, FGD-gypsum consumption, insurance, maintenance and labour. The amortization costs are calculated as follows (Anon, 1998):

(3) Q = C * (r(1+r)^n^) / ((1+r)^n^-1)

where Q is the periodic payment, C is the investment of the NH_3_ stripping and scrubbing unit and the filter press (1.85 M€), r is the interest rate fixed at 3% and n is the depreciation of the mentioned units fixed at 10 years. The considered cost of electricity was 0.15 € kWh^−1^. Use of FGD-gypsum amounted to 12 € t^−1^. Insurance, maintenance and labour costs were calculated as 4% of the total investment.

Revenues were calculated assuming that ammonium sulphate and liming substrate were traded with the following nutrient market values: 770 € t^−1^ N, 550 € t^−1^ S and 60 € t^−1^ CaO (GNS, personal communication). The market value of ammonium sulphate solution and liming substrate were thus calculated respectively at around 67 € t^−1^ and 47 € t^−1^. The premium for heat valorisation envisaged by the German EGG was included as well (0.02 € kWh^−1^).

## 3 Results & discussion

### 3.1 Characterisation of process streams

Chicken manure fed to co-AD system from January until April 2019 was characterised for TN, TP and TK. In this period, manure was analysed about 300 times when delivered at Benas farm, giving the following results: TN 21 ± 5.7 g kg^−1^ FW, NH_4_–N 4.4 ± 2 g kg^−1^ FW, TP 5.9 ± 2 g kg^−1^ FW, TK 10 ± 3.6 g kg^−1^ FW.

Composition and physico-chemical parameters for the process intermediates and end-points are presented in Table 1. The pH values of fertiliser suspension, ammonium sulphate and liming substrate ranged from 7.6 to 7.9. Internally in the stripping system, the highest pH value (9.9 ± 0.18) was measured in the stripping effluent. During the vacuum stripping phase, waste heat from CHP engines was used to increase the temperature of the stripper influent from 47 ± 0.64 °C to 76 ± 1.7 °C, which enhanced the shift of NH_4_^+^ towards volatile NH_3_ in digestate (Siegrist et al., 2013). Moreover, the increment of pH conditions in the stripping towers was achieved without any base addition due to simultaneous sequestration of CO_2_. At neutral pH, inorganic C is mainly present in the form of HCO_3_^−^. At high pH, CO_2_ is formed from HCO_3_^−^ following (Eq. (4)) and (Eq. (5)) (Cohen and Kirchmann, 2004).

(4) HCO_3_- + H_2_O → H_2_CO_3_ + OH^−^

(5) H_2_CO_3_ → CO_2_↑ + H_2_O

**Table 1:** Physicochemical composition (mean ± standard deviation, n = 3) on fresh weight (FW) basis of solid and liquid products within the Benas process flows: digestate, liquid fraction digestate after screw press (LF digestate), solid fraction digestate after screw press (SF digestate), stripper influent, FGD-gypsum, stripper effluent, fertiliser suspension after stripping, ammonium sulphate, liming substrate.

Products	PH	EC (mS cm^−1^)	DW (g kg^−1^ FW)	OM (g kg^−1^ FW)	TN (g kg^−1^ FW)	NH_4_–N (g kg^−1^ FW)
Digestate	8.4 ± 0.09	31 ± 2.8	118 ± 2.1	82 ± 9	7.9 ± 2.1	4.4 ± 0.43
LF digestate	8.4 ± 0.1	31 ± 2	100 ± 10	65 ± 8.3	7.4 ± 2	4.3 ± 0.81
SF digestate	8.6 ± 0.3	5 ± 0.2	252 ± 5.5	189 ± 10	8.7 ± 1.2	4.4 ± 0.94
Stripper influent	8.5 ± 0.12	29 ± 0.96	119 ± 3.5	82 ± 2.3	8.2 ± 1.7	4.5 ± 0.48
Stripper effluent	9.9 ± 0.18	16 ± 0.46	126 ± 5.5	86 ± 3.9	5.8 ± 0.89	1.8 ± 0.29
FGD-Gypsum	7.6 ± 0.091	2 ± 0.18	750 ± 30	–	0.26 ± 0.1	0.12 ± 0.056
Fertiliser suspension	7.6 ± 0.083	166 ± 20	391 ± 38	–	38 ± 2.3	38 ± 5.8
Ammonium sulphate	7.8 ± 0.037	223 ± 15	224 ± 11	–	46 ± 3.6	46 ± 2.5
Liming substrate	7.9 ± 0.045	15 ± 1.5	695 ± 32	–	15 ± 2	15 ± 2.6
Products	TP (g kg^−1^ FW)	TK (g kg^−1^ FW)	TS (g kg^−1^ FW)	TCa (g kg^−1^ FW)	TMg (g kg^−1^ FW)	TNa (g kg^−1^ FW)
Digestate	1.6 ± 0.26	6.9 ± 0.73	1.2 ± 0.085	4.2 ± 0.86	0.75 ± 0.15	0.66 ± 0.12
LF digestate	1.5 ± 0.31	6.7 ± 0.8	1.2 ± 0.12	3.7 ± 1.2	0.69 ± 0.25	0.64 ± 0.16
SF digestate	2.2 ± 0.22	5.5 ± 2.3	1.6 ± 0.27	4.4 ± 0.91	1.3 ± 0.19	0.58 ± 0.16
Stripper influent	1.8 ± 0.13	7.1 ± 0.79	1.2 ± 0.083	4.2 ± 0.83	1 ± 0.22	0.66 ± 0.14
Stripper effluent	1.9 ± 0.12	7.7 ± 0.97	1.3 ± 0.1	4.8 ± 1.1	1.1 ± 0.19	0.72 ± 0.15
FGD-Gypsum	0.21 ± 0.085	0.39 ± 0.13	159 ± 7	218 ± 9	0.28 ± 0.12	0.17 ± 0.02
Fertiliser suspension	0.057 ± 0.0074	0.13 ± 0.046	50 ± 8.4	65 ± 29	0.1 ± 0.061	0.057 ± 0.02
Ammonium sulphate	0.0033 ± 0.0018	0.0039 ± 0.0017	58 ± 0.81	1.2 ± 0.42	0.0067 ± 0.0015	0.0039 ± 0.0022
Liming substrate	0.19 ± 0.049	0.4 ± 0.24	32 ± 12	225 ± 39	0.33 ± 0.16	0.19 ± 0.09

Concentrations of macronutrients varied for all liquid and solid streams (Table 1). With regards to digestate, LF digestate and SF digestate, there were no significant differences in pH, ranging between 8.4 and 8.6. The higher pH value of SF digestate compared to LF digestate was probably due to the higher content of Ca (respectively 4.4 ± 0.91 g kg^−1^ FW and 3.7 ± 1.2 g kg^−1^ FW), in accordance with Bachmann et al. (2016).

SF digestate was characterised by relatively high DW and OM values (respectively 252 ± 5.5 g kg^−1^ FW and 189 ± 10 g kg^−1^ FW), due to the greater quantity of undigested fibres. The lower water content of SF digestate translates into easier storage and transport compared to digestate and LF digestate. The ratio of the DW content in LF digestate over the DW content in digestate (DW_LF_:DW_digestate_) of the screw press monitored in this study resulted to be 0.84, suggesting a poor solids separation. This is in agreement with Akhiar et al. (2017) results, who observed a DW_LF_:DW_digestate_ in screw press above 0.8. SF digestate contained the highest concentration of TP (2.2 ± 0.22 g kg^−1^ FW), confirming the lower solubility of P in water. The N:P ratio confirmed preferential segregation of P in SF digestate: the ratio decreased from 4.9 in digestate and LF digestate to 3.9 in SF digestate. Compared to LF digestate, SF digestate was richer on average in TN, TP, TS, TCa and TMg. Despite TK, TNa (and their ionic forms K^+^ and Na^+^) and NH_4_–N, are very soluble, thus dissolved in the liquid phase (Masse et al., 2005), their fractionation in LF and SF digestate was similar. As result, NH_4_–N, TK and TNa concentrations were comparable in LF and SF digestate. This is probably due to the low separation efficiency of the screw press. Nevertheless, compared to SF digestate, LF digestate displayed a higher NH_4_–N:TN ratio (0.59). With almost 60% of TN in the form of mineral N, LF digestate represents an interesting substitute for mineral N fertilisers (Tambone and Adani, 2017).

Concerning the ammonium sulphate production steps, the highest DW content was measured in liming substrate, (695 ± 32 g kg^−1^ FW). Ammonium sulphate showed the highest value of TN (46 ± 3.6 g kg^−1^ FW), entirely present in mineral form, which explains the high conductivity of the solution (223 ± 15 mS cm^−1^). Hence, the highest NH_4_–N:TN ratio was found in ammonium sulphate (1), whilst the lowest was found in the stripper effluent (0.32). Compared to stripper influent, stripper effluent exhibited higher concentrations of TP, TK, TS, TCa, TMg and TNa. The upconcentration of these elements was due to the removal of water under vacuum conditions in the stripping towers. As expected, only TN and NH_4_–N were higher in the stripper influent compared to the N depleted stripper effluent. The highest and lowest amounts of TCa were respectively in the two final products: liming substrate (225 ± 39 g kg^−1^ FW) and ammonium sulphate (1.2 ± 0.42 g kg^−1^ FW). Similarly, ammonium sulphate displayed modest values of TP (0.0033 ± 0.0018 g kg^−1^ FW), TK (0.0039 ± 0.0017 g kg^−1^ FW), TMg (0.0067 ± 0.00015 g kg^−1^ FW) and TNa (0.0039 ± 0.0022 g kg^−1^ FW). Finally, ammonium sulphate solution showed the highest content of TS (58 ± 0.81 kg^−1^ FW).

### 3.2 Mass balances, separation and recovery efficiencies

**Fig. 3:**
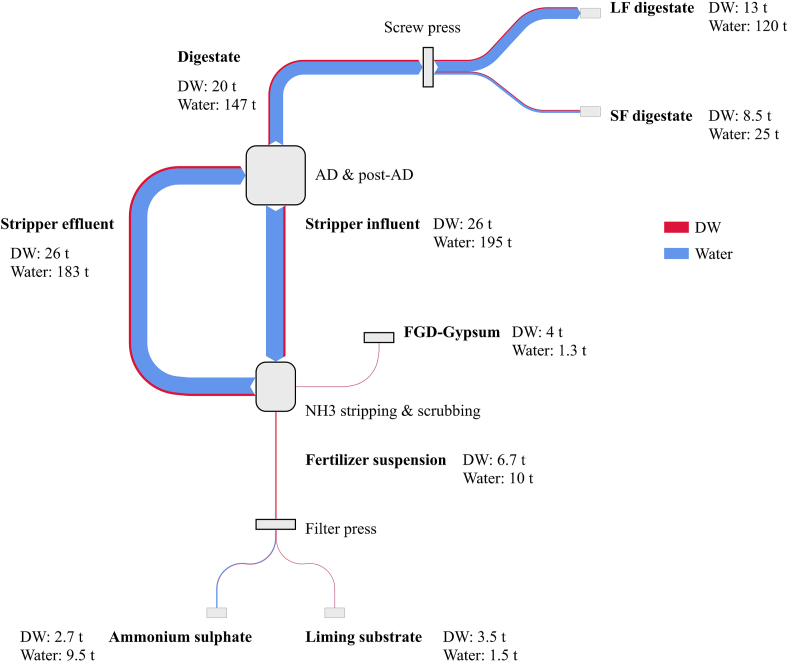
Mass balance for dry weight (DW) and water expressed in t d^−1^ of solid and liquid products within the Benas process flows: digestate, liquid fraction digestate after screw press (LF digestate), solid fraction digestate after screw press (SF digestate), stripper influent, FGD-gypsum, stripper effluent, fertiliser suspension, ammonium sulphate, liming substrate.

**Fig. 4:**
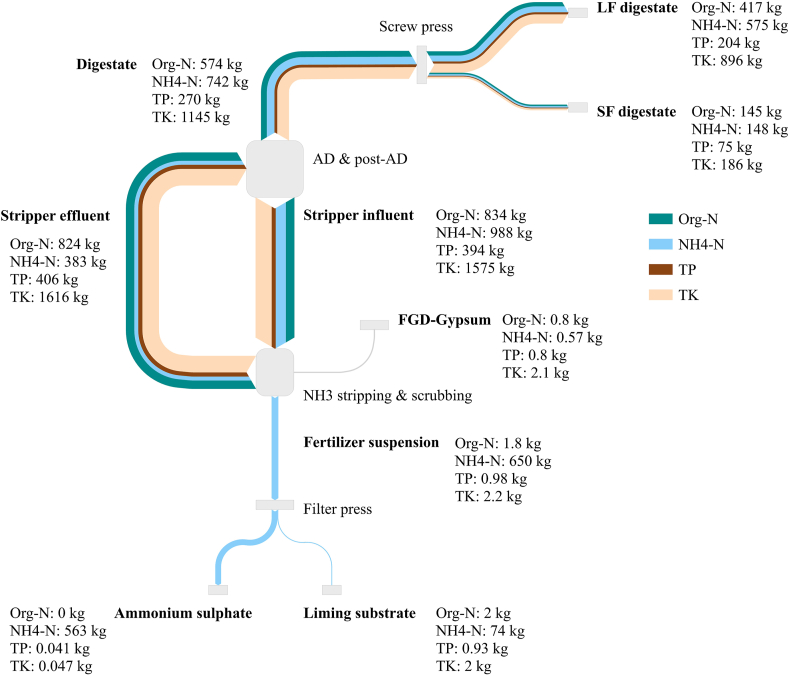
Mass balance for organic nitrogen (Org-N), ammonium nitrogen (NH_4_–N), total phosphorus (TP), total potassium (TK) expressed in kg d^−1^ of solid and liquid products within the Benas process flows: digestate, liquid fraction digestate after screw press (LF digestate), solid fraction digestate after screw press (SF digestate), stripper influent, FGD-gypsum, stripper effluent, fertiliser suspension, ammonium sulphate, liming substrate.

**Fig. 5:**
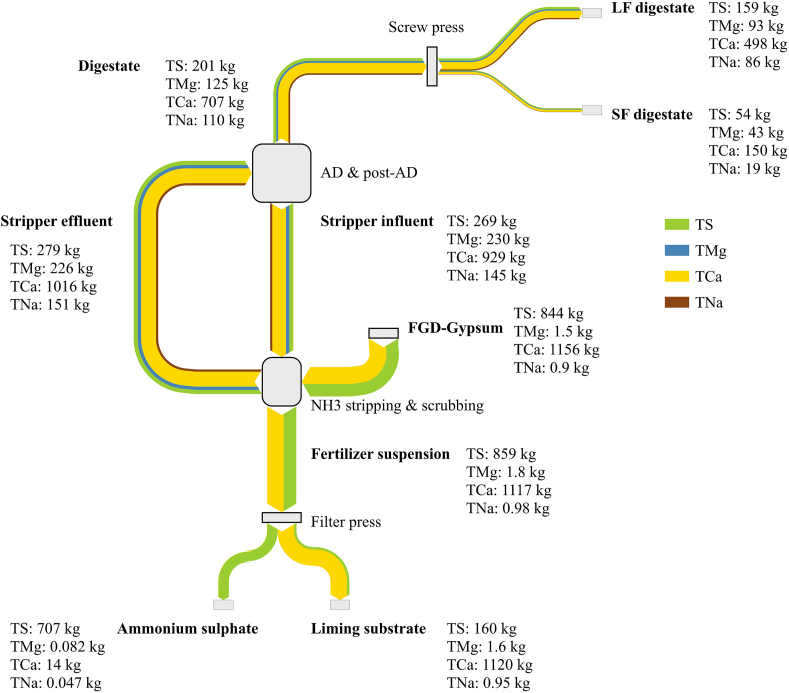
Mass balance for total sulphur (TS), total magnesium (TMg), total calcium (TCa) and total sodium (TNa) expressed in kg d^−1^ of solid and liquid products within the Benas process flows: digestate, liquid fraction digestate after screw press (LF digestate), solid fraction digestate after screw press (SF digestate), stripper influent, FGD-gypsum, stripper effluent, fertiliser suspension, ammonium sulphate, liming substrate.

Material flows of DW and water are shown in Fig. 3, whereas flows of Org-N, NH_4_–N, TP and TK are presented in Fig. 4 and flows of TS, TMg, TCa and TNa are depicted in Fig. 5.

On average 222 t d^−1^ of stripper influent was processed in the side stream NH_3_ stripping and scrubbing unit, where NH_3_ is transferred from the liquid to gas phase. The gas phase, rich in NH_3_, entered into a reactor where an aqueous suspension containing 5.3 t d^−1^ FGD-gypsum was spread to form 17 t d^−1^ of a fertiliser suspension containing ammonium sulphate and liming substrate. The suspension was further processed using a filter press to obtain 12 t d^−1^ of 22% ammonium sulphate solution and 5 t d^−1^ of liming substrate. 210 t d^−1^ of stripper effluent was recirculated back the co-AD installation to be further fermented. Finally, 167 t d^−1^ of digestate from the storage tank were mechanically separated into 34 t d^−1^ of SF digestate and 133 t d^−1^ of LF digestate by means of a screw press. In the same monitoring period, the co-AD plant generated on average 63 t d^−1^ of biogas (52,628 Nm^3^ d^−1^; 53% CH_4_ and 47% CO_2_).

Concerning the NH_3_ stripping unit, 222 t d^−1^ of influent were fed to the NH_3_ stripper, resulting in 210 t d^−1^ of stripper effluent. This corresponded to a water flow of 195 t d^−1^ in the stripper influent and 183 t d^−1^ in the stripper effluent (Fig. 3). The amount of water removed by the vacuum stripper represented 6.1%, which allowed the process to generate ammonium sulphate solution without the addition of external water sources. Consequently, this resulted in an increased concentration of all elements in the stripper effluent. During the stripping phase, all elements were approximately in equilibrium (<10% accumulation or reduction). The stripping unit was fed with 1,822 kg d^−1^ of TN, of which 54% was present in the form of NH_4_–N and 46% in the form of Org-N. 36% of TN contained in the stripper influent was recovered as fertiliser suspension, corresponding to an NH_4_–N recovery efficiency of 66% (650 kg d^−1^). Reversely, the remaining part of TN was recirculated back to the co-AD as stripper effluent mainly in the form of Org-N (Fig. 4). In AD systems, the excess of NH_3_ can have an inhibiting effect on methanogenic microorganisms when reaching toxic levels, compromising the anaerobic fermentation process. Effective dilution of chicken manure can be achieved by adjusting the C:N ratio with suitable feedstock or by recirculating N depleted digestate. Ghyselbrecht et al. (2018) investigated the biogas production of pretreated digestate via NH_3_ stripping as compared to untreated digestate. Results indicated that after eight days, biogas yields of stripped digestate was 36% higher, which was allocated to the lower NH_3_ concentration in the digester. It seems possible that preventing NH_3_ from reaching harmful concentrations at Benas co-AD was achieved by diluting the incoming feedstock with recirculated N depleted digestate.

Regarding the NH_3_ washing step, the fertiliser suspension was observed to consist predominantly out of NH_4_–N as expected, but it was observed to also contain small amounts of Org-N. Since during the stripping process only NH_4_–N is removed from the treated slurry, the presence of Org-N in the fertiliser suspension can be attributed to the addition of FGD-gypsum (0.26 ± 0.10 g kg^−1^ FW of TN and 0.12 ± 0.061 g kg^−1^ FW of NH_4_–N) as scrubbing medium. Due to its competitive price, H_2_SO_4_ is the most commonly used acid during NH_3_ washing step. Nevertheless, other agents such as nitric acid, boric acid, organic acids and gypsum have also been tested to recover NH_3_ from the gas phase, generating scrubbing salts (Abouelenien et al., 2009; Jamaludin et al., 2018; Mohammed-Nour et al., 2019; Sigurnjak et al., 2019). To our knowledge, Benas is the first AD plant that installed an industrial vacuum NH_3_ stripping and scrubbing unit relying on gypsum. FGD-gypsum used at Benas was collected from a coal power plant and traces of elements other than Ca and S are therefore expected to be present. FGD-gypsum is comparable to natural gypsum with reference to heavy metal content, thus making it a promising product in agricultural applications (Watts and Dick, 2014). The presence of limited amounts of TP, TK, TMg and TNa in the fertiliser suspension and its components (ammonium sulphate and liming substrate) reflects, in fact, the chemical composition of FGD-gypsum (Table 1).

Proceeding to the filter press step, the amount of TN, NH_4_–N, TK, TP, TS, TCa, TMg and TNa in ingoing and outgoing streams was roughly balanced. Fractionation of TN, NH_4_–N and TS was mainly associated with the liquid ammonium sulphate (respectively 85%, 87% and 82%). In contrast, TP (95%), TK, (97%), TCa (100%) TMg (92%) and TNa (97%) were mostly recovered in liming substrate (Fig. 4, Fig. 5). Overall, 31% of TN (57% of NH_4_–N) contained in the stripper influent was recovered in the form of ammonium sulphate (46 ± 3.6 g kg^−1^ of TN), while 4.2% of TN (7.5% of NH_4_–N) was collected in the form of liming substrate (15 ± 2.0 g kg^−1^ of TN). On average, 12 t d^−1^ of 22% ammonium sulphate solution were generated, amounting to 2.5 kg of NH_4_–N recovered per tonne of digestate processed in the stripping and scrubbing unit.

Recovery of mineral N via stripping and scrubbing described in this study resulted in similar or higher values than recoveries reported in the literature (Table 2). Sigurnjak et al. (2019) described a biogas plant located in Northern Italy, where the installed side stream NH_3_ stripping and scrubbing unit allowed for the recovery of about 10% of the input TN waste fed to the digestion process. TN recovery from the influent stripper reaches values up to 35%, in the form of 36% ammonium sulphate (74 g kg^−1^ TN) (this paper). Bolzonella et al. (2018) monitored a digestate processing system where the liquid fraction of digestate obtained via screw press and settler separation entered in a stripping and scrubbing unit. In this case, 22% of the TN contained in the stripper influent was collected as ammonium sulphate. Overall, the TN recovery reported from initial digestate was 17%, resulting in an ammonium sulphate solution at 26 g kg^−1^ TN. Differently from the two previous cases, Ledda et al. (2013) described a digestate processing cascade where digestate was first mechanically separated and the liquid fraction digestate was subsequently processed in a membrane filtration system. Finally, the reverse osmosis (RO) centrate was treated in a stripping and scrubbing system where 17–33% of TN (35–53% of NH_4_–N) present in digestate was recovered in the form of 22–31% ammonium sulphate (51–61 g kg^−1^ TN) and 1.5–2.5% of TN (3–4% of NH_4_–N) was recovered as lime residue. In comparison to different digestate processing technologies, such as the vibratory shear enhanced processing (VSEP) membrane filtration, full scale NH_3_ stripping and scrubbing facilities seem to achieve lower recoveries. In fact, TN recovered in RO centrate using VSEP technology was almost 55% of TN contained in digestate (Vaneeckhaute et al., 2012). Nevertheless, the TN content of recovered RO centrate (7.3 ± 1.6 kg^−1^ FW) is lower compared to scrubbing salts recycled via stripping and scrubbing.

**Table 2:** Anaerobic digestion feedstock, process conditions of the stripping unit (pH, temperature, stripping gas), scrubbing media, energy consumption (electricity and heat), recovery efficiencies as ammonium sulphate (AS) and liming substrate (LS), AS production cost and AS market value of full scale stripping and scrubbing installations, integrated at anaerobic digestion plants.

AD feedstock	Stripping conditions			Scrubbing medium dosage	Energy consumption		Recovery efficiency from digestate		AS production cost	AS market value	Reference
	pH	Temperature °C	Stripping gas	kg kg^−1^ N recovered	kWh_el_ kg^−1^ N recovered	kWh_th_ kg^−1^ N recovered	TN	NH_4_–N		€ m^−3^ (% TN)	
Cow manure, pig effluents, energy crops	>9 Lime addition	70 - 80 (air)	Air	3.5 kg H_2_SO_4_	12	–	17% AS	23% AS	5.4 € m^−3^ digestate	30 (6% TN)	Bolzonella et al. (2018)
cow manure	12–12.5 Lime addition	Ambient	Air	H_2_SO_4_	–	–	17% AS 1.5% LS	35% AS 3% LS	4.2 € m^−3^ digestate	50 (6–8% TN)	Ledda et al. (2013)
swine manure	12–12.5 Lime addition	Ambient	Air	H_2_SO_4_	–	–	33% AS 2.5% LS	53% AS 4% LS	4.2 € m^−3^ digestate	50 (6–8% TN)	Ledda et al. (2013)
sewage sludge, food waste	–	60–80	Biogas	7.3 kg H_2_SO_4_ (50% solution)	5	–	35% AS	–	–	–	Sigurnjak et al. (2019), Systemic project
corn silage, chicken manure, other crops	8.5–9.9 CO_2_ stripping	47–76	Vacuum	8.4 kg CaSO_4_.2H_2_O (75% DW)	3.8	59	31% AS 4.2% LS	57% AS 7.5% LS	5.8 € t^−1^ digestate	35 (4.6% TN)	This study

Regarding the screw press separation step, digestate was separated into a liquid and a solid fraction. Although the DW content of SF digestate (252 ± 5.5 kg^−1^ FW) was higher compared to LF digestate (100 ± 10 kg^−1^ FW), DW was mainly associated with the LF digestate (67%) and only 43% of the DW content was associated with the SF digestate (Fig. 3). This is because SF digestate represented only 20% of digestate mass after screw press separation, whereas LF digestate the remaining 80%. All elements (TN, NH_4_–N, TP, TK, TS TMg, TCa and TNa) were in equilibrium between ingoing and outgoing streams (Fig. 4, Fig. 5) and all nutrients were mainly drained with the LF digestate. These findings are comparable with results obtained with screw press separators by Bachmann et al. (2016) and Popovic et al. (2012). Even though their studies were conducted on different digestate feedstock (57% dairy slurry and 43% maize) or substrate (pig manure), a DW accumulation in the solid fraction was registered between 40 and 46%. Møller et al. (2002) demonstrated that a considerable amount of small particles is accumulated in the liquid fraction as they pass through the filter pores. Since N, P and K are likely to be in the liquid phase or associated with small particles (Hjorth et al., 2011), higher amounts of these elements are expected to migrate predominantly in the liquid fraction, following results from this study. A small reduction of Org-N (2.1%) can be attributed to the degradation of OM carried out by biological activity during storage of digestate prior separation. Minor losses of NH_4_–N (2.7%) were also identified, probably due to N escaping the system in the form of N gas (N_2_) or NH_3_. Conversely, slightly larger outgoing mass flows of TP, TS and TMg could be explained by the retention of SF digestate in the screw press and consequent release in the subsequent separations.

### 3.3 Energy balance

The energy balance was computed for the year 2019, where about 65,542 t of digestate were generated from the AD of organic feedstock and 68,561 t of digestate were processed in the side stream NH_3_ stripping and scrubbing unit.

During 2019, Benas co-AD generated about 20 Mm^3^ of biogas, of which about 60% was converted into electrical and thermal energy, while 40% was upgraded to biomethane. The electrical energy generated at Benas corresponded to 307 kWh_el_ t^−1^ feedstock (427 kWh_el_ t^−1^ digestate produced), whereas the thermal energy produced amounted to 277 kWh_th_ t^−1^ feedstock (389 kWh_th_ t^−1^ digestate). Finally, biomethane generated accounted for 417 kWh t^−1^ feedstock (575 kWh t^−1^ digestate).

The electricity required by the digestate processing cascade was around 9.3 kWh_el_ t^−1^ digestate treated, which is about 2.3% of the total electricity generated on-site (Table 3). Stripping of NH_3_ was the most energy-intensive step (7.5 kWh_el_ t^−1^ digestate), followed by the NH_3_ adsorbing system, which required 0.81 kWh_el_ t^−1^ digestate. Screw press and filter press consumed about 0.5 kWh_el_ t^−1^ digestate each one. Concerning the thermal energy requirements, 37% of the heat generated by the CHP engines was utilised in the stripping columns (139 kWh_th_ t^−1^ digestate). Vaneeckhaute et al. (2017) reported an energy use around 1.54–12 kWh_el_ m^−3^ and 62–69 kWh_th_ m^−3^. As such, electricity consumption for ammonium sulphate production at Benas (stripping and scrubbing system and filter press) is in line with literature data, whereas heat requirement is higher. In 2019, approximately 161 t of TN were removed with the NH_3_ stripping and scrubbing system to generate 3,545 t of biobased ammonium sulphate solution (22%). This corresponds respectively to 3.8 kWh_el_ and 59 kWh_th_ kg^−1^ N recovered as 22% ammonium sulphate. Tampio et al. (2016) extensively reviewed the electricity consumption of stripping and scrubbing installations operating on different substrates, including animal manure, digestate and urine. Electrical energy requirements described ranged between 0.8 and 28 kWh_el_ kg^−1^ N recovered. Furthermore, Bolzonella et al. (2018) calculated 12 kWh_el_ kg^−1^ N recovered, while the biogas plant located in Northern Italy previously described required about 5 kWh_el_ kg^−1^ N recovered (Systemic project). The production of biobased ammonium sulphate at Benas required 3.8 kWh_el_ kg^−1^ N recovered, following literature results (Table 2).

**Table 3:** Energy (electricity and heat) consumption of each unit of the digestate processing cascade. In brackets, the percentage of energy required over the total generated.

	Electricity kWh_el_ t^−1^ digestate	Heat kWh_th_ t^−1^ digestate
NH_3_ stripping	7.5 (1.8%)	139 (37%)
NH_3_ scrubbing	0.81 (0.20%)	
Filter press	0.5 (0.12%)	
Screw press	0.5 (0.12%)	
Total	9.3 (2.3%)	139 (37%)

### 3.4 Economic assessment of ammonium sulphate production

The CBA for the production of biobased ammonium sulphate solution was performed including as process steps the NH_3_ stripping and scrubbing and the filter press (Table 4). Revenues and costs from the AD process and digestate separation via mechanical separation were therefore excluded from the analyses. The CBA was computed for the year 2019, where about 68,561 t of digestate were processed to generate 3,545 t of ammonium sulphate solution.

**Table 4:** Economic assessment of the production of biobased ammonium sulphate generated at Benas. All costs and benefits are expressed in € per tonne of digestate processed in the NH_3_ stripping and scrubbing system.

	Cost € t^−1^ digestate	Benefit € t^−1^ digestate
Amortised capital cost	3.2	
Electrical energy	1.3	
FGD-gypsum	0.23	
Insurance, maintenance, labour	1.1	
Ammonium sulphate revenue		3.5
Liming substrate revenue		0.78
Heat valorisation		2.8
Total	5.8	7.0

CAPEX included capital costs for the NH_3_ stripping and scrubbing plant (including costs for the storage tank of ammonium sulphate) and for the filter press. OPEX involved electrical energy requirements, FGD-gypsum consumption, insurance, maintenance and labour costs, whereas the total investment amounted to 1.85 M € (depreciation 10 years at 3% interest rate). The total cost amounted to 5.8 € t^−1^ digestate, in accordance with Vaneeckhaute et al. (2017) who reported an overall cost for industrial stripping and scrubbing installations ranging between 2.0 and 8.1 € m^−3^, depending on the operational conditions. Our findings are comparable also with results from Bolzonella et al. (2018) and Ledda et al. (2013), who estimated a total cost of 5.4 and 4.2 € m^−1^ digestate treated, respectively (Table 2).

Economic benefits were calculated around 7 € t^−1^ digestate and included the avoided costs for the purchase of synthetic mineral N fertilisers (3.5 € t^−1^ digestate) as well as liming substrates (0.78 € t^−1^ digestate). Additionally, incentives from the valorisation of heat generated by the CHP engines were included. Benefits related to a higher biogas yield resulting from stripper effluent recirculation, as well as reduced volumes of digestate storage and transport were both not taken into account. The values of ammonium sulphate and liming substrate were calculated respectively at 67 and 47 € t^−1^ according to the following nutrient market values: 770 € t^−1^ N, 550 € t^−1^ S and 60 € t^−1^ CaO (GNS, personal communication). However, the reported ammonium sulphate economic value of 67 € t^−1^ holds only if ammonium sulphate is used on Benas own fields as replacement for fossil based N fertilisers. According to GNS, trade of ammonium sulphate solution outside Benas farm, would decrease the N fertiliser commercial value by 50%, thereby not exceeding 35 € t^−1^ (GNS, personal communication). Similarly, Bolzonella et al. (2018) predicted a value of 30 € m^−1^ for a 30% ammonium sulphate solution (6% TN). This value is higher than that reported by Laureni et al. (2013), who estimated the price of ammonium sulphate solution (6% TN) at around 21 € t^−1^ (0.35 € kg^−1^ N) (Table 2). According to Ledda et al. (2013), a commercial value of around 50 € t^−1^ of ammonium sulphate can be reached by farms through the subscription to the Fertilisers Producers Register (Dl. 217/2006), and the product registration to the conventional fertilisers register. The difference might be explained by the different local market. According to Vaneeckhaute et al. (2017), higher revenues from ammonium sulphate trade are expected when the particle size of crystals is larger, more precisely for ammonium sulphate with a coarse fraction of 80% > 1.8 mm. As such, the production of “granular” scrubbing salts seem to be an attractive option to boost the marketability of these fertilising commodities.

### 3.5 Fertiliser quality of ammonium sulphate

Table 5 summarises the characteristics that scrubbing salts would have to be classified as RENURE products (Huygens et al., 2020) and/or as inorganic fertiliser, under the Fertilising Product Regulation (EU) 1009/2019. Concerning the product characteristics of RENURE fertilisers, JRC refers to RENURE as “*any N containing substance fully or partially derived from livestock manure through processing under conditions that can be used in areas with water pollution by N following the same provisions applied to N containing chemical fertilisers as defined in the Nitrates Directive (91/*676/EEC*), while providing adequate agronomic benefits to enhance plant growth*”. Ammonium sulphate recovered at Benas complied with all the requirements to fulfil the criteria indicated by JRC's RENURE products. Specifically, TOC:TN ratio, Cu and Zn amounts are below the minimum indicated. Furthermore, the NH_4_–N:TN ratio is above 90%.

**Table 5:** Legal limits on product characteristics of different fertilisers defined by the Fertilising Product Regulation (EU) 1009/2019 and Joint Research Centre (JRC) RENURE products (Huygens et al., 2020), in comparison with biobased ammonium sulphate generated at Benas (FW: fresh weight; DW: dry weight).

Fertiliser type	Nutrient content g kg^−1^ FW	TN g kg^−1^ FW	SO_3_ g kg^−1^ FW	TOC g kg^−1^ FW	TOC:TN	NH_4_–N:TN (%)	Cu mg kg^−1^ DW	Zn mg kg^−1^ DW
PFC 1(C)(I)(b)(ii) (Fertilising Product Regulation)	≥70^a^	≥15	≥7.5	≤10			≤600	≤1500
RENURE product (JRC)					≤3^b^	≥90^b^	≤300	≤800
Ammonium sulphate (Benas)	185	46 ± 3.9	137 ± 9.3	0.35 ± 0.12	0.0076	100	0.1 ± 0.11	0.47 ± 0.12

a The sum of nutrient contents (TN, P_2_O_5_, K_2_O, MgO, CaO, SO_3_, Na_2_O) shell be at least 70 g kg^−1^ FW.

b For RENURE products either the threshold for TOC:TN ratio or NH_4_–N:TN ratio should be met.

Ammonium sulphate solution examined in this study reached all the compositional requirements necessary to be classified as a compound liquid inorganic macronutrient fertiliser (PFC 1(C)(I)(b)(ii)) of the Fertilising Product Regulation. TN is 3-fold higher than the minimum content proposed by the EU, whereas the declared nutrient content is double; sulphur trioxide (SO_3_) is about 20 times the minimum quantity. Conversely, TOC, Cu and Zn are largely below the maximum allowed. Finally, the biobased N fertiliser generated at Benas biogas plant contains N entirely in mineral form, and as such represent an interesting alternative for the substitution of synthetic mineral N fertilisers. This is in agreement with the Circular Economy Action Plan, which stirs the substitution of nutrients from primary raw materials with recycled nutrients.

RENURE products can represent a good solution to substitute chemical N fertilisers; nevertheless simpler and less expensive solutions should also be considered in the light of the most recent scientific evidence about the effect of digestate and derived products (i.e. LF digestate) on plant production (Riva et al., 2016; Tambone et al., 2017).

Also agricultural and environmental negative aspects can be mentioned about the land application of scrubbing salts, such as the high EC and sulphate concentration. These factors may be detrimental for soil quality in terms of salt accumulation, decreasing in turn crop production. Moreover, the high NH_4_–N:TN ratio (1) may lead to harmful NH_3_ emissions that can, nevertheless, be mitigated by direct injection followed by ploughing (Vaneeckhaute et al., 2013). This is in agreement with SAFEMANURE criteria, which indicates that NH_3_ emission during RENURE products application should be minimised by immediate incorporation or comparable procedures.

## 4 Conclusions

A nutrient recovery system, including a vacuum NH_3_ stripping and scrubbing system relying on FGD-gypsum allowed for the recovery of 57% of NH_4_–N present in digestate, in the form of ammonium sulphate solution. This in turn allows to produce biogas from high N feedstock without suffering inhibitory effects from the NH_3_. The characteristics of recovered ammonium sulphate (22% solution), whose production cost was calculated around 5.8 € t^−1^, would fit the proposed criteria as a RENURE product and as inorganic fertiliser, enabling the reuse of N derived from manure as inorganic biobased N fertiliser in the European market in replacement of mineral (fossil resource based) N fertiliser.

## CRediT authorship contribution statement

**C. Brienza:** Conceptualization, Methodology, Software, Formal analyses, Investigation, Data curation, Writing – original draft, Writing – review & editing. **I. Sigurnjak:** Conceptualization, Writing – review & editing, Visualization, Supervision. **T. Meier:** Formal analyses, Investigation, Writing – review & editing. **E. Michels:** Supervision, Project administration. **F. Adani:** Writing – review & editing, Funding acquisition. **O. Schoumans:** Conceptualization, Writing – review & editing, Funding acquisition. **C. Vaneeckhaute:** Writing – review & editing. **E. Meers:** Conceptualization, Resources, Writing – review & editing, Supervision, Funding acquisition.

## Declaration of competing interest

The authors declare that they have no known competing financial interests or personal relationships that could have appeared to influence the work reported in this paper.
